# Effectiveness of a childhood obesity prevention programme delivered through schools, targeting 6 and 7 year olds: cluster randomised controlled trial (WAVES study)

**DOI:** 10.1136/bmj.k211

**Published:** 2018-02-07

**Authors:** Peymane Adab, Miranda J Pallan, Emma R Lancashire, Karla Hemming, Emma Frew, Tim Barrett, Raj Bhopal, Janet E Cade, Alastair Canaway, Joanne L Clarke, Amanda Daley, Jonathan J Deeks, Joan L Duda, Ulf Ekelund, Paramjit Gill, Tania Griffin, Eleanor McGee, Kiya Hurley, James Martin, Jayne Parry, Sandra Passmore, K K Cheng

**Affiliations:** 1 Institute of Applied Health Research, University of Birmingham, Birmingham, UK; 2 School of Clinical and Experimental Medicine, University of Birmingham; Birmingham, UK; 3 Edinburgh Migration, Ethnicity and Health Research Group, Usher Institute of Population Health Sciences and Informatics, The University of Edinburgh, UK; 4 Nutritional Epidemiology Group, School of Food Science and Nutrition, University of Leeds, Leeds, UK; 5 Warwick CTU, University of Warwick, Warwick, UK; 6 School of Sport, Exercise and Rehabilitation Sciences, University of Birmingham, Birmingham, UK; 7 Cambridge MRC Epidemiology Unit, Cambridge, UK; 8 Department of Sports Medicine, Norwegian School of Sport Sciences, Oslo, Norway; 9 Birmingham Community Healthcare NHS Trust, Birmingham, UK; 10 Services for Education, Birmingham, UK

## Abstract

**Objective:**

To assess the effectiveness of a school and family based healthy lifestyle programme (WAVES intervention) compared with usual practice, in preventing childhood obesity.

**Design:**

Cluster randomised controlled trial.

**Setting:**

UK primary schools from the West Midlands.

**Participants:**

200 schools were randomly selected from all state run primary schools within 35 miles of the study centre (n=980), oversampling those with high minority ethnic populations. These schools were randomly ordered and sequentially invited to participate. 144 eligible schools were approached to achieve the target recruitment of 54 schools. After baseline measurements 1467 year 1 pupils aged 5 and 6 years (control: 28 schools, 778 pupils) were randomised, using a blocked balancing algorithm. 53 schools remained in the trial and data on 1287 (87.7%) and 1169 (79.7%) pupils were available at first follow-up (15 month) and second follow-up (30 month), respectively.

**Interventions:**

The 12 month intervention encouraged healthy eating and physical activity, including a daily additional 30 minute school time physical activity opportunity, a six week interactive skill based programme in conjunction with Aston Villa football club, signposting of local family physical activity opportunities through mail-outs every six months, and termly school led family workshops on healthy cooking skills.

**Main outcome measures:**

The protocol defined primary outcomes, assessed blind to allocation, were between arm difference in body mass index (BMI) z score at 15 and 30 months. Secondary outcomes were further anthropometric, dietary, physical activity, and psychological measurements, and difference in BMI z score at 39 months in a subset.

**Results:**

Data for primary outcome analyses were: baseline, 54 schools: 1392 pupils (732 controls); first follow-up (15 months post-baseline), 53 schools: 1249 pupils (675 controls); second follow-up (30 months post-baseline), 53 schools: 1145 pupils (621 controls). The mean BMI z score was non-significantly lower in the intervention arm compared with the control arm at 15 months (mean difference −0.075 (95% confidence interval −0.183 to 0.033, P=0.18) in the baseline adjusted models. At 30 months the mean difference was −0.027 (−0.137 to 0.083, P=0.63). There was no statistically significant difference between groups for other anthropometric, dietary, physical activity, or psychological measurements (including assessment of harm).

**Conclusions:**

The primary analyses suggest that this experiential focused intervention had no statistically significant effect on BMI z score or on preventing childhood obesity. Schools are unlikely to impact on the childhood obesity epidemic by incorporating such interventions without wider support across multiple sectors and environments.

**Trial registration:**

Current Controlled Trials ISRCTN97000586.

## Introduction

Excess weight in childhood is a global problem, affecting around 41 million children under the age of 5 years.^1^ In addition to physical and psychosocial health consequences in these early years, childhood excess weight is an important predictor of obesity in adulthood,^2^ with additional adverse health and economic^3^ effects. In the UK around a quarter of children have excess weight at school entry (age 4 or 5 years).^4^ The proportion of very overweight children doubles during the subsequent six years (from approximately 9% to 19%),^4^ highlighting this period as critical for preventive action.

Systematic reviews of childhood obesity prevention studies suggest that school based interventions may be effective in reducing the proportion of children with excess weight.^5^ ^6^ Heterogeneity of study design and interventions precludes conclusions about which combination of components are likely to be most effective. Nevertheless, overall, longer duration, multicomponent interventions, targeting school curriculums and food and physical activity environments, providing teacher support, and extending activities to the home and community were more likely to be associated with positive results. However, trials to date have had several methodological weaknesses that limit recommendations for widespread implementation.^5^ In particular, few previous trials reported longer term outcomes, subgroup effects, or cost effectiveness.

We report the results of the West Midlands ActiVe lifestyle and healthy Eating in School children (WAVES) study; a cluster randomised controlled trial evaluating an intervention that aims to prevent excess weight in primary school children. The trial dealt with the main limitations identified in previous research: use of the Medical Research Council framework for complex intervention development and evaluation^7^; a sample size large enough to detect clinically significant differences in adiposity; a comprehensive process evaluation; assessment of longer term effects, using a range of adiposity and psychosocial measures; and an objective measure of physical activity.

## Methods

### Trial design and eligibility

This was a school based, cluster randomised, controlled trial evaluating the effectiveness of a complex obesity prevention intervention on primary school children’s body mass index (BMI) z scores at 15 and 30 months after baseline measurements (3 and 18 months post-intervention completion).^8^

Primary schools in the West Midlands, UK, within 35 miles of the study centre were eligible for inclusion (n=980). The region includes a multiethnic population from diverse socioeconomic backgrounds living in rural and urban areas. We excluded schools with fewer than 17 year 1 (aged 5 and 6 years) pupils (minimum cluster size) or schools in “special measures” (unlikely to have capacity to contribute to study). Within participating schools, all children in year 1 at recruitment were eligible for inclusion.

### Interventions and intervention development

Irrespective of whether children participated in measurements, intervention delivery was at school class level to all eligible children and their families.

The development process of the WAVES study intervention commenced in 2005. We summarised intervention components incorporated in previous childhood obesity prevention trials (70 included studies within eight systematic reviews) in relation to setting, target behaviour, and type of activity. To help prioritise intervention components we then conducted focus groups with parents, school staff, and local health, government, and community members. The discussions considered the perceived importance and feasibility of implementation of techniques (eg, reward behaviours, role model, exposure to opportunities for physical activity), activities (eg, education materials, cooking workshops), and particular settings (eg, school curriculum, community setting). We checked prioritised ideas against available local resources, and the intervention package was formed with input from an expert group of professionals. Thus we balanced the prioritised intervention components (eg, role models to influence behaviour, or family campaigns) with the resources that were readily available in our setting (eg, the Villa Vitality programme described later). This intervention comprised activities within two broad aims: increasing children’s physical activity levels through school and home and supporting the development of health behaviour skills in families through activity based learning.^9^ The intervention was further refined following a feasibility study.^10^ That study showed that the proposed measurements could be completed successfully (measurements obtained for 574 out of 606 children with consent (95%) at baseline) and that loss to follow-up two years after baseline was at an acceptable level (follow-up measurements obtained for 83% and 86% of children in control and intervention schools, respectively). The feasibility study was not powered to investigate intervention outcomes, but the direction of effect was in favour of the intervention for most outcomes. In particular, children in the intervention arm compared with control arm had significantly lower adjusted BMI z scores at follow-up (−0.15 kg/m^2^, 95% confidence interval −0.27 to −0.03). Table 1 provides details of the finalised intervention.

**Table 1:** Summary of WAVES study intervention programme

Intervention component *and fit with stakeholder prioritisation*	Who delivers and details	When delivered	Intended participants
**Aim 1: increase children’s physical activity levels**			
Daily opportunity for additional 30 minutes of moderate to vigorous physical activity in bouts of >5 minutes through classroom or playground routines *Extracurricular intervention to increase school day physical activity*	Class teacher guided through choice of four resources^8^ to assist in delivery. Type of activity and timing of delivery tailored by teacher according to class circumstances	Every school day in year 2, within school time (≥15 minutes to be outside of school breaks)	Children
Brightly coloured information sheets: a) signposting local facilities and opportunities for family based physical activity outside school b) including motivational messages and ideas for being active at home *Community initiatives and facilities*	Detailed information on opportunities and facilities for family based physical activity in locality, prepared by researchers in consultation with school staff, distributed to families through school	After randomisation and term 1 in year 2	Children and their families
Villa Vitality programme (iconic sport institution to provide role model and motivation). Three sessions over six weeks, interspersed by weekly family “challenges” and a class project *Role models* *Family campaigns*	Indoor and outdoor sessions led by Villa community coaches, highlighted ways in which children could incorporate physical activity into their daily lives Family challenges included a pledge to be active for at least an hour a day Class teachers worked with their class to develop a song on healthy lifestyles	Six week programme any time during year 2. Coaching sessions: two half days at football ground; one hour in school	Children with family support for weekly challenges
**Aim 2: improve children’s dietary intake**			
Cooking skills workshops to increase knowledge and equip families with skills to prepare healthier food (increase fruit, vegetable, and fibre intake, and reduce fat and sugar intake) *Parent education sessions to confer skills* *Family activities*	Teacher provided with training and resources to deliver workshops and two or three short lessons on healthy eating before each workshop. Parents invited to accompany children during workshops, led by teachers, where they practised skills (eg, chopping, grating, peeling, mixing) to prepare a meal. Written information sent home after each session	Three workshops (breakfast, lunch/snacks, evening meal); one each term during year 2	Children and parents
Villa Vitality programme (iconic sport institution to provide role model and motivation). Two sessions over six weeks, interspersed by weekly family “challenges” and a class project. Sessions designed to reinforce healthy eating messages and skills from cooking workshops *Role models* *Family campaigns*	Villa community staff provided interactive sessions on healthy eating and supervised practical preparation of a healthy meal over the two stadium visit days (six weeks apart) Family challenges: swap a snack, drink more water, eat a healthy breakfast every day, eat five portions of fruit and vegetables every day, and cook a healthy family meal Class teachers worked with their class to develop a song on healthy lifestyles	Six week programme any time during year 2, with two half day sessions at football ground	Children with family support for weekly challenges

### WAVES study intervention and its delivery

The intervention components, delivered over 12 months, targeted the home and school environment. The target group, based on findings from the feasibility study, was year 2 children (aged 6 or 7 years) and their families. Several behaviour change strategies were employed to encourage increased physical activity and improved diet quality. School staff were provided with training and resources for intervention delivery. A termly family newsletter reinforced messages delivered through the various intervention components. The intervention programme (summarised in table 1) comprised four overlapping components:

(1) Thirty minutes of additional moderate to vigorous physical activity on each school day—at least 15 minutes to be outside of break times, although class teachers customised timing of delivery and exact activities undertaken according to their class circumstances, supported by resources supplied as part of the study. Class teachers selected two preferred resources out of four offered and were taken through each selected resource and its detailed delivery materials by a researcher

(2) Termly cooking workshops during school time, which parents were invited to attend to participate in with their child and that were preceded by short classroom sessions for the children. School staff responsible for implementation (with the exception of two schools where equivalent training was delivered in school by the same researcher) attended a one day training session. To minimise teacher preparation time and ensure delivery of consistent nutritional messages, the presentation and interactive activity materials, together with take home information sheets and suggested lesson and workshop plans were provided, but timing of sessions and how parents were involved was left to the discretion of teachers

(3) A six week programme (Villa Vitality) developed to encourage healthy eating and increase physical activity and delivered by staff from an iconic sporting institution. School classes spent two days undertaking activities (indoor based movement routines, using dance mats, ball skills session, interactive nutritional sessions, and an opportunity to practise cooking skills) at an English premier league football club, separated by a six week period during which teachers were asked to spend curriculum time working on a class project and involving children and their parents with weekly health challenges. The teacher customised the elements undertaken in school supported by a school visit from a member of staff from Villa Vitality

(4) Information sheets signposting children and their families on ways to be active over the summer (identical for all schools) and physical activity opportunities in their local area (school specific sheets produced by the study team and checked before distribution by the school).

### Comparator intervention

Schools allocated to the comparator arm continued with ongoing year 2 health related activities. In addition, we provided citizenship education resources, excluding topics related to healthy eating and physical activity.

### Outcomes

The primary outcome for clinical effectiveness specified in our analysis plan and trial protocol was the difference in BMI z scores between arms at 15 and 30 months. Table 2 summarises the trial protocol prespecified secondary outcome measures. At trial registration, the secondary outcomes of waist circumference, sum of four skinfolds, and body fat percentage were included as primary outcomes. All outcomes were assessed at 15 and 30 months post-baseline measures (3 and 18 months post-intervention). Further details on the methods, including standardised operating procedures for all primary and secondary outcome measurements, are available in the final report of the WAVES study, available through the National Institute for Health Research website (www.journalslibrary.nihr.ac.uk).

**Table 2:** Summary of measurements undertaken within WAVES study and their associated outcome variables

Measurements	Time points				Instrument	No of measures at each time point	Method of assessment	Outcome variables*
	Baseline	1st follow-up	2nd follow-up	3rd follow-up				
Weight	Yes	Yes	Yes	Yes	Tanita bioimpedance monitor (Tanita SC-331S; Tanita, Tokyo, Japan)	Once	Barefoot and in light clothing	Body mass index (BMI) z score Overweight or obese (BMI ≥85th centile or ≥95th centile (both using UK 1990 BMI reference curves for children^33^)
Height	Yes	Yes	Yes	Yes	Leicester height measure	Twice (third measure if difference >0.4 cm)†	Barefoot and in light clothing	
Demographic data (sex and date of birth)	Yes	No	No	No	Parent questionnaires	NA	Parent report and school records	
Body fat %	Yes	Yes	Yes	Yes	Tanita bioimpedance monitor (Tanita SC-331S; Tanita, Tokyo, Japan)	Once	Barefoot and in light clothing using two limb (legs) bioelectrical impedance technology	Body fat %
Waist circumference (to nearest 0.1 cm)	Yes	Yes	Yes	Yes	Flexible, non-stretch, cloth tape measure	Twice (third measure if difference >0.4 cm)†	Measured at iliac crest	Waist circumference z score using UK 1990 BMI reference curves for children^33^
Skinfold thickness (biceps, triceps, subscapular, suprailiac, and thigh)	Yes	Yes	Yes	Yes	Holtain Tanner/Whitehouse Skinfold Caliper (Holtain, UK)	Twice (third measure if difference >0.4 mm)^‡^	Measured on non-dominant side	Sum of four skinfolds§ (biceps, triceps, suprailiac, and subscapular)
Dietary intake	Yes	Yes	Yes	Yes	Child And Diet Evaluation Tool (CADET) (a validated 115 item 24 hour food tick list^22^ completed for seven distinct time periods)	Once (24 hours)	Completed by trained researchers in school, and parent/carer at home (with instructional DVD)	Dietary daily total energy intake (kJ in 24 hours), fat, sugar, fibre (g/day), and fruit and vegetable intake (g/day and portions)
Physical activity	Yes	Yes	Yes	No	Actiheart (Cambridge Neurotechnology, Papworth, UK)	Once (worn continuously for five days, including a weekend)	Fitted in school by trained researcher	Daily physical activity energy expenditure (kJ/kg body weight/day)¶, and time spent being sedentary and undertaking at least moderate intensity activity (min/24 hours) assessed by Actiheart
Blood pressure	Yes	Yes	Yes	Yes	Automated oscillometric monitor (BpTRU BPM-100, British Columbia, Canada)	Twice (third measure if error reading, or if one value outside normal range)**	Three minutes seated rest before and between readings	Systolic and diastolic blood pressure
Quality of life	Yes	Yes	Yes	Yes	Pediatric quality of life inventory (PedsQL)	NA	Researcher administered questionnaire	Self reported health related quality of life
Social acceptance	Yes	Yes	Yes	Yes	Kidscreen-52 health questionnaire for children and young people	NA	Researcher administered questionnaire	Social acceptance
Body image dissatisfaction	Yes	Yes	Yes	Yes	Child’s body image scale (CBIS)	NA	Researcher administered questionnaire (score derived from sex specific 7 point child’s body image scale)	Body image dissatisfaction
Demographic data (date of birth, sex, ethnicity, postcode (proxy measure for deprivation))	Yes	No	No	No	Parent questionnaires	NA	Parent report and school records	Sex, ethnicity, deprivation (index of multiple deprivation)

Measurements were carried out by trained research staff using standard protocols.

* Primary outcomes (from trial protocol)=difference in BMI z scores between arms at first follow-up and second follow-up, all other outcomes are trial protocol secondary outcomes but some of the measures of obesity (proportion of children overweight/obese between arms, waist circumference z score, sum of four skinfolds, and body fat %) were included as primary outcomes in the trial registration.

† Where two values were within ≤0.4 cm, a definitive measurement value was calculated as the average of the two. For individuals with three values recorded, a definitive measurement value was calculated as the average of the closest pair (within ≤0.4 cm) or average of all three readings (if there were no two closest readings, but the differences between values were ≤0.4 cm). When none of the three values were within 0.4 cm of each other no definitive measurement value was calculated.

‡ Where two values were within ≤0.4 mm, a definitive measurement value was calculated as the average of the two. For individuals with three values recorded, a definitive measurement value was calculated as the average of the closest pair (within ≤0.4 mm) or average of all three readings (if there were no two closest readings, but the differences between values were ≤0.4 mm). When none of the three values were within 0.4 cm of each other no definitive measurement value was calculated.

§ Skinfold thickness was measured at five different sites (biceps, triceps, thigh, suprailiac, and subscapular), as detailed in the protocol. Compared with the other sites, however, the children found the measurement of thigh skinfold thickness more intrusive, resulting in a lower level of data availability for this compared with the other sites. The skinfold thickness summary measure was therefore calculated excluding the thigh measurement.

¶ Children with less than 24 hours of valid data were excluded. In addition, to ensure representation across the whole 24 hour period, for those with 24 hours of valid data, only those with a distribution of at least six hours in each quadrant of the day (morning; 3 am-9 am, noon; 9 am-3 pm, afternoon; 3 pm-9 pm, and midnight; 9 pm-3 am) were included.

** Readings with a systolic or diastolic, or both, value 20 mm hg above the 99.6th centile of the UK age specific and sex specific reference data^34^ were excluded as implausible values. Pairs of readings for which an error message was returned for either the systolic or diastolic value were excluded. Subsequent to these exclusions, systolic and diastolic values that remained were treated independently. For individuals with only one systolic or diastolic value this was taken as the definitive measurement value. For individuals with two remaining systolic or diastolic values the definitive measurement value was taken as the average of the two values. For individuals with three remaining systolic or diastolic values, provided there was a closest pair of values, the definitive measurement value was taken as the average of these two values, or, in instances of no closest pair, the average of all three values.

### Implementation

The trial statistician (KH) undertook sampling and subsequent randomisation, and the trial coordinator (ERL) recruited schools. To enable subgroup analysis we stratified schools by ethnic mix of pupils, and we used a weighted random sampling strategy to increase the selection likelihood (3:1) of schools with a higher minority ethnic population. Using this method, we selected 200 schools, which were ordered using a random number generator and sequentially invited to participate. To allow measurement of a large number of children in a limited timeframe within study resources, we recruited and randomised the schools into two groups (27 schools in each group), one year apart. Parental informed consent was sought and verbal assent from the children was obtained for all measurements undertaken.

### Participant assessment and data collection procedures

Baseline assessment took place when children were at the end of year 1 (aged 5 or 6 years). Outcome assessments using identical procedures were undertaken at 15 months (first follow-up) and 30 months (second follow-up) post-baseline (aged 7 or 8, and 8 or 9 years, respectively). In schools recruited in the first year (group 1), we further assessed at 39 months (third follow-up) post-baseline (aged 9 or 10 years), but this was not possible for schools recruited in the second year (group 2) within the trial timetable. We collected data from school records, direct assessment of participating children in school, and parent questionnaires distributed at the time of pupil measurements. Trained research staff undertook assessments using standardised protocols and validated instruments, as detailed in the protocol^8^ and summarised in table 2.

### Sample size

Sample size was based on the primary outcome (BMI z score), taking into account repeated measures (estimated correlation between measures=0.9), varying cluster size (assuming mean 25 (SD 23) cluster size), and likely estimates of the intraclass correlation coefficient (0 to 0.04). To detect a clinically meaningful difference of 0.25 BMI z score^11^ between intervention and comparator groups with 90% power, a two sided α of 0.05, and estimated pupil dropout rate of 20%, we needed a follow-up sample of 1000 children from 50 schools. Allowing for school drop-out of 8%, we recruited 54 schools. This sample size also provides more than 80% power to detect a 0.125 difference in BMI z score (clinically important difference for prevention^12^) and an approximately 7% difference in the change of proportion of children who are overweight or obese from baseline to follow-up in control compared with intervention schools.

### Randomisation

A blocked balancing algorithm was used to randomise participating schools to intervention or comparator arms. Schools were randomly allocated according to a randomisation scheme, which minimised imbalance^13^ on several characteristics: percentage of pupils eligible for free school meals (measure of deprivation), proportion of pupils from South Asian, black African-Caribbean, white, or other ethnic groups, and school size. We randomised the first 27 schools (group 1) within the first block. A year later we randomly allocated the remaining 27 schools (group 2) in a similar way, but conditioning on the allocations that had already been made in group 1.

To ensure concealment of allocation we carried out randomisation after baseline measurements. Sessional researchers blind to arm allocation mainly undertook further data collection. Supplementary figure 1 summarises the timeline for trial processes.

### Statistical analysis

Analyses of all outcomes were by intention to treat and are reported at 15, 30, and 39 months after baseline (3, 18, and 27 months after the end of the intervention). For the primary analyses (complete case analysis), we used mixed linear regression models for all continuous outcomes (eg, BMI z score) and Poisson mixed regression for binary outcomes to allow estimation of adjusted risk differences consistent with CONSORT guidelines. To accommodate any non-normality of the outcomes, we transformed data when necessary and when such transformation improved the model. The baseline adjusted model included the baseline measurement and treatment arm as the independent variables, and to account for the clustered nature of the sample, school as the random effect. We also report models further adjusted for prespecified baseline school and child level covariates. Planned subgroup analyses, using interaction tests, examined whether any intervention effects differed by ethnicity, sex, socioeconomic status, baseline weight status, and fidelity of implementation.

Sensitivity analyses included using multiple imputation (using chained equations) for missing values for each outcome, exploring cluster heterogeneity by period (group 1 versus group 2 schools), and methods of adjusting for missing baseline variables to maximise use of available data and heterogeneity of the intraclass correlation coefficient in intervention and control arms. Additional details on the statistical methods are available in the final report, available through the National Institute for Health Research website (www.journalslibrary.nihr.ac.uk).

We set the level of statistical significance at 0.05 (two sided) for the primary outcomes (see table 2) and at 0.01 for all other outcomes. Analyses were carried out in Stata 13^14^ and REALCOM-impute^15^ software.

Because of the timelines of recruitment and outcome assessments, there was no opportunity for interim analyses. The trial steering committee maintained assessment of data quality and completion.

#### Process evaluation

We used a variety of methods for assessment of intervention delivery and process, including interviews with teachers; parent and child focus groups; head teacher, class teacher, and parental questionnaires; teacher logbooks; and direct observation of sessions by researchers.^16^ With the exception of the signposting sheets for which there was no variation in implementation between schools, we used a consensus method for each of the other three intervention components to allocate schools a score on a 5 point Likert scale for each dimension of the process evaluation (fidelity and adherence; reach, dose, and exposure; recruitment, quality, and participant responsiveness). Context and information on programme differentiation influence all of these and were therefore also considered throughout this score allocation process. We then ranked schools by total score, and grouped the schools to reflect low, medium, or high intervention implementation. A detailed report on the method used to synthesise the process evaluation data is published elsewhere.^17^

#### Changes to methods from trial registration stage

The trial registration was submitted before the practical planning for the trial had started. Some aspects were subsequently altered in the development of the trial protocol, but the trial registration was not updated and therefore does not incorporate these changes. Supplementary table 1 summarises all changes between trial registration and trial protocol. In particular, at the early planning stages for the trial (and before the start of baseline measurements) the investigator team modified the primary outcomes from those specified at trial registration. To increase power to detect change and for consistency and comparability with previous trials, we changed the primary outcome for clinical effectiveness from the binary variable specified in the registry (of difference in proportion of children categorised as overweight or obese between arms) to the continuous outcome specified in the protocol of difference in BMI z scores between arms. Concurrently this binary variable and the additional anthropometric measurements included as primary outcomes at trial registration were specified as secondary outcomes. The reporting of the trial is in keeping with the published protocol,^8^ which was submitted before the start of data analysis, but any differences between what is reported and the trial registration information are specified in both the text and the tables.

### Patient and public involvement

Public involvement was a key feature of the early phases of trial development and feasibility testing before this main trial. Intervention development was informed by detailed consultation with parents, teachers, and other school staff. The intervention was further refined and the process for measuring outcomes tested and adapted by asking the children, parents, and teachers about their experiences during the feasibility study. Measures of wellbeing and body dissatisfaction were included as outcomes based on their perceived importance among school staff. Our research team includes an education advisor at the Health Education Service, who has regular contact with schools and advised on school and participant recruitment. No patients were involved in this trial.

## Results

**Fig 1:**
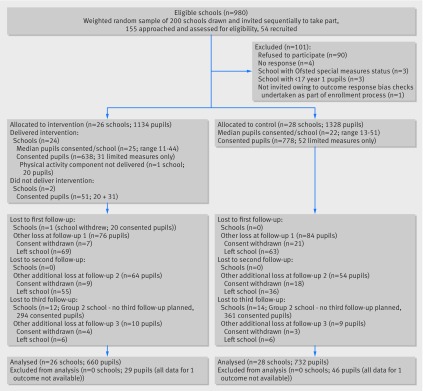
Flow of school recruitment and trial arm allocation

Figure 1 shows the flow of schools and pupils during the trial. Among 2462 eligible pupils from 54 participating schools at baseline, parental consent for baseline measurements was obtained from 1467 (59.6%). Recruitment took place between April and May 2011 (group 1 schools and pupils) and from January to May 2012 (group 2 schools and pupils). Table 3 summarises the baseline characteristics. Although school characteristics were balanced between the two groups, there was baseline imbalance at the pupil level, with children in the control arm compared with intervention arm more likely to be male (52.7% *v* 49.2%), from generally less deprived households (mean index of multiple deprivation score 37.6 *v* 39.8), less likely to be overweight (mean BMI z score 0.15 *v* 0.23), more likely to consume five portions of fruit and vegetables daily (64.8% *v* 59.8%), and more likely to achieve at least 60 minutes of moderate to vigorous physical activity daily (49.6% *v* 46.4%).

**Table 3:** Baseline characteristics of school pupils participating in the WAVES study overall and by trial arm

Characteristics	Intervention arm	Control arm	Total
**Demographic**	n=662	n=735	n=1397
Mean (SD) age (years); not known	6.3 (0.3); 27	6.3 (0.3); 43	6.3 (0.3); 70
Sex:	n=689	n=778	n=1467
Male	339 (49.2)	410 (52.7)	749 (51.1)
Female	350 (50.8)	368 (47.3)	718 (48.9)
Ethnicity:	n=676	n=775	n=1451
White British	297 (43.9)	361 (46.6)	658 (45.3)
South Asian	221 (32.7)	222 (28.6)	443 (30.5)
Black African-Caribbean	62 (9.2)	53 (6.8)	115 (7.9)
Other	96 (14.2)	139 (17.9)	235 (16.2)
Not known*	13	3	16
Deprivation fifth†:	n=670	n=769	n=1439
1 (most deprived)	392 (58.5)	398 (51.8)	790 (54.9)
2	120 (17.9)	154 (20.0)	274 (19.0)
3	72 (10.7)	74 (9.6)	146 (10.1)
4	65 (9.7)	54 (7.0)	119 (8.3)
5 (least deprived)	21 (3.1)	89 (11.6)	110 (7.6)
Median (interquartile range) deprivation score†; not known*	39.8 (21.9-52.7); 19	37.6 (17.9-48.8); 9	38.9 (20.1-49.5); 28
**Anthropometric**			
BMI z score:	n=660	n=732	n=1392
Mean (SD) BMI z score; not known	0.23 (1.2); 29	0.15 (1.2); 46	0.19 (1.2); 75
Height (cm):	n=664	n=732	n=1396
Mean (SD); not known:	118.6 (5.6); 25	118.2 (5.4); 46	118.4 (5.5); 71
Waist circumference, z score:	n=589	n=670	n=1259
Mean (SD); not known:	0.77 (1.2); 100	0.66 (1.3); 108	0.71 (1.3); 208
Sum of skinfolds (mm)‡	n=540	n=597	n=1137
Median (interquartile range); not known	28.6 (23.3-35.4); 149	28.1 (23.0-36.6); 181	28.4 (23.1-36.1); 330
Body fat %:	n=660	n=716	n=1376
Mean (SD); not known	21.3 (5.4); 29	21.0 (5.2); 62	21.1 (5.3); 91
Weight status§:	n=660	n=732	n=1392
Underweight (≤2nd centile)	20 (3.0)	20 (2.7)	40 (2.9)
Healthy weight (>2nd and <85th centiles)	495 (75.0)	562 (76.8)	1057 (75.9)
Overweight (≥85th and <95th centiles)	61 (9.2)	63 (8.6)	124 (8.9)
Obese (≥95th centile)	84 (12.7)	87 (11.9)	171 (12.3)
Not known*	29	46	75
**24 hour dietary intake**	n=562	n=625	n=1187
Median (interquartile range) energy (kJ/24 hrs); not known	6904 (5865-8054); 127	6911 (5804-7964); 153	6907 (5829-8002); 280
≥5 portions of fruit and vegetables:	n=562	n=625	n=1187
Yes	336 (59.8)	405 (64.8)	741 (62.4)
No	226 (40.2)	220 (35.2)	446 (37.6)
Not known*	127	153	280
**Physical activity**	n=492	n=560	n=1052
Mean (SD) physical activity energy expenditure (kJ/kg/day); not known	96.4 (23.2); 197	94.1 (24.4); 218	95.2 (23.8); 415
≥60 mins MVPA/24 hours:	n=491	n=557	n=1048
Yes	228 (46.4)	276 (49.6)	504 (48.1)
No	263 (53.6)	281 (50.4)	544 (51.9)
Not known*	198	221	419
**Psychological**	n=663	n=721	n=1384
Median (interquartile ranage) PedsQL total score; not known	71.7 (60.9-82.6); 26	73.9 (60.9-82.6); 57	71.7 (60.9-82.6); 83

MVPA=moderate to vigorous physical activity; PedsQL=pediatric quality of life inventory.

* Not included in denominator for calculation of percentages.

† Index of multiple deprivation.

‡ Subscapular, suprailiac, biceps, and triceps skinfolds.

§ Based on UK 1990 reference centile curves and applying cut-offs used for population monitoring.

### Primary outcomes

The primary outcomes are also reported in the trial protocol (table 4). At 15 months the mean BMI z score was non-significantly lower in the intervention arm compared with control arm: mean difference −0.075 (95% confidence interval −0.183 to 0.033, P=0.18) in baseline adjusted models (n=1197, 86% of those with baseline BMI z score available) and −0.077 (−0.191 to 0.037, P=0.19) in further adjusted (n=837, 60% of those with baseline BMI z score available) models. At 30 months the mean difference was smaller and remained non-significant (−0.027, −0.137 to 0.083; P=0.63) in the baseline adjusted model (n=1094, 79% of those with baseline BMI z score available).

**Table 4:** Adjusted differences for body mass index (BMI) z score between control and intervention groups at first, second, and third follow-up

Follow-up	No of participants (No in intervention arm)	Mean (SD) BMI z scores			Mean difference (95% CI), P value	
		Intervention arm*	Control arm†		Intervention *v* control (baseline adjusted)‡	Intervention *v* control (further adjusted)§
15 months	n=1197¶ (n=556) baseline adjusted; n=837¶ (n=393) further adjusted	0.34 (1.34)	0.23 (1.27)		−0.075 (−0.183 to 0.033), 0.18	−0.077 (−0.191 to 0.037), 0.19
30 months	n=1094¶ (n=505baseline adjusted; n=772¶ (n=359) further adjusted	0.42 (1.34)	0.31 (1.32)		−0.027 (−0.137 to 0.083), 0.63	−0.042 (−0.163 to 0.080), 0.50
39 months	n=467** (n=232baseline adjusted; n=345** (n=173) further adjusted	0.49 (1.37)	0.63 (1.22)		−0.204 (−0.396 to −0.013), 0.04; (99% CI −0.456 to 0.048)	−0.177 (−0.386 to 0.033), 0.03; (99% CI −0.386 to 0.033)

* Baseline, all participants 0.23 (1.24); baseline, group 1 school participants only 0.29 (1.24).

† Baseline, all participants 0.15 (1.20); baseline group 1 school participants only 0.28 (1.12).

‡ Adjusted for baseline outcome.

§ Adjusted for baseline outcome, baseline pupil level covariates: (sex, ethnicity, deprivation (index of multiple deprivation score for home postcode), 24 hour total energy intake, physical activity energy expenditure) and baseline school level covariates (size (number of pupils on roll), % school population South Asian, % school population black African-Caribbean, % free school meal eligibility).

¶ Includes group 1 and group 2 school participants.

** Includes group 1 school participants only.

### Secondary outcomes

The secondary outcomes are as reported in the trial protocol and trial registration information, unless stated otherwise (table 5).

**Table 5:** Adjusted differences for secondary outcomes (anthropometric, diet, physical activity, and psychosocial) between control and intervention arm at first and second follow-up

Outcomes: No at FU1/No at FU2 (No in intervention arm)	Mean (SD)/median (interquartile range)/No (%)								Mean difference (99% CI) or risk difference (99% CI), P value								
	Intervention arm				Control arm				Intervention *v* control (baseline adjusted)*					Intervention *v* control (further adjusted)†			
	Baseline	FU1	FU2		Baseline	FU1	FU2		FU1		FU2			FU1		FU2	
**Obese‡**																	
FU1: n=1197 (n=556, baseline adjusted), 837 (n=393, further adjusted); FU2: n=1094 (n=505, baseline adjusted), 772 (n=359, further adjusted)	84 (12.73)	93 (16.20)	108 (20.61)		87 (11.89)	100 (14.81)	112 (18.04)		−0.036 (−0.073 to 0.019)	0.074	−0.004 (−0.050 to 0.057)	0.837		−0.007 (−0.046 to 0.045)	0.676	0.020 (−0.030 to 0.086)	0.336
**Obese or overweight‡§**																	
FU1: n=1197 (n=556, baseline adjusted), 837 (n=393, further adjusted; FU2: n=1094 (n=505, baseline adjusted), 772 (n=359, further adjusted)	145 (21.97)	165 (28.75)	176 (33.59)		150 (20.49)	167 (24.74)			−0.013 (−0.075 to 0.071)	0.655	0.002 (−0.068 to 0.093)	0.948		0.000 (−0.064 to 0.087)	0.994	0.004 (−0.062 to 0.087)	0.892
Sum of four skinfolds (mm)‡¶**																	
FU1: n=902 (n=421, baseline adjusted), 683 (n=323, further adjusted); FU2: n=724 (n=334, baseline adjusted), 560 (n=262, further adjusted)	28.55 (23.30-35.43)	31.48 (24.57-43.65)	34.70 (25.5-49.95)		28.10 (23.00-36.60)	29.40 (23.63-41.67)	31.93 (24.00-48.90)		0.366 (−0.322 to 1.054)	0.170	0.644 (−0.067 to 1.356)	0.020		0.417 (−0.384 to 1.219)	0.180	0.532 (−0.268 to 1.333)	0.087
**Waist z score‡**																	
FU1: n=1069 (n=490, baseline adjusted), n=796 (n=368, further adjusted); FU2: n=923 (n=414, baseline adjusted), n=703 (n=320, further adjusted)	0.77 (1.24)	1.05 (1.36)	1.15 (1.25)		0.66 (1.25)	0.87 (1.32)	0.90 (1.35)		0.026 (−0.229 to 0.281)	0.794	0.103 (−0.087 to 0.293)	0.163		0.019 (−0.166 to 0.205)	0.789	0.068 (−0.133 to 0.269)	0.383
**Body fat %‡**																	
FU1: n=1169 (n=553, baseline adjusted), n=822 (n=391, further adjusted); FU2: n=1051 (n=495, baseline adjusted), n=747 (n=354, further adjusted)	21.30 (5.35)	21.79 (6.73)	22.52 (7.48)		20.95 (5.22)	20.87 (6.30)	21.58 (7.26)		0.040 (−0.942 to 1.021)	0.917	0.344 (−0.629 to 1.317)	0.362		0.048 (−0.999 to 1.095)	0.906	0.166 (−0.992 to 1.324)	0.712
**Energy intake (kJ/24 hrs)**††																	
FU1: n=978 (n=449, baseline adjusted), n=803 (n=369, further adjusted); FU2: n=895 (n=401, baseline adjusted), n=729 (n=331, further adjusted)	6904 (5865-8054)	7152 (6107-8376)	7656 (6436-9118)		6911 (5804-7964)	7074 (5963-8233)	7817 (6748-9212)		61.531 (−305.536 to 428.597)	0.666	−139.552 (−570.798 to 291.693)	0.405		30.988 (−348.629 to 410.604)	0.833	−273.658 (−724.284 to 176.967)	0.118
**Fat intake (g/24 hrs)^††^**																	
FU1: n=978 (n=449, baseline adjusted), n=803 (n=369, further adjusted); FU2: n=895 (n=401, baseline adjusted), n=729 (n=331, further adjusted)	56.08 (45.39-69.28)	60.95 (47.32-71.98)	65.66 (51.81-79.88)		54.74 (44.75-67.58)	57.36 (46.87-70.15)	67.41 (54.59-81.08)		1.426 (−2.291 to 5.143)	0.323	−1.943 (−6.629 to 2.742)	0.285		1.260 (−2.336 to 4.857)	0.367	−2.740 (−7.652 to 2.171)	0.151
**Free sugars intake (g/24 hrs)**																	
FU1: n=978 (n=449, baseline adjusted), n=803 (n=369, further adjusted); FU2: n=895 (n=401, baseline adjusted), n=729 (n=331, further adjusted)	76.63 (31.01)	72.05 (33.03)	74.50 (32.18)		76.13 (30.88)	75.31 (32.88)	81.21 (35.16)		−4.329 (−12.781 to 4.124)	0.187	−7.886 (−18.488 to 2.716)	0.055		−5.636 (−12.285 to 1.014)	0.029	−9.220 (−19.032 to 0.592)	0.015
**Fibre intake (g/24 hrs)**††																	
FU1: n=978 (n=449, baseline adjusted), n=803 (n=369, further adjusted); FU2: n=895 (n=401, baseline adjusted), n=729 (n=331, further adjusted)	11.00 (8.80-13.68)	11.76 (9.41-14.62)	12.44 (10.01-15.47)		11.35 (8.99-13.95)	11.77 (9.18-14.46)	12.77 (10.44-15.66)		0.013 (−0.767 to 0.793)	0.965	−0.163 (−1.162 to 0.837)	0.675		0.008 (−0.914 to 0.930)	0.982	−0.461 (−1.499 to 0.577)	0.253
**Fruit and vegetable intake (g/24 hrs**)‡‡																	
FU1: n=978 (n=449, baseline adjusted), n=803 (n=369, further adjusted); FU2: n=895 (n=401, baseline adjusted), n=729 (n=331, further adjusted)	226.92 (132.00-330.09)	200.23 (91.79-315.28)	218.06 (115.60-348.41)		247.58 (157.25-341.40)	201.84 (116.16-316.56)	219.28 (116.54-341.33)		−2.875 (−33.148 to 27.399)	0.807	14.195 (−29.969 to 58.360)	0.408		−5.652 (−41.150 to 29.847)	0.682	14.598 (−34.821 to 64.018)	0.447
**≥5 portions of fruit and vegetables**§§																	
FU1: n=978 (n=449, baseline adjusted), n=803 (n=369, further adjusted); FU2: n=895 (n=401, baseline adjusted), n=729 (n=331, further adjusted)	336 (59.79)	244 (48.13)	253 (55.85)		405 (64.80)	297 (49.09)	317 (56.41)		−0.014 (−0.111 to 0.109)	0.753	0.012 (−0.090 to 0.135)	0.789		0.004 (−0.075 to 0.098)	0.900	0.002 (−0.096 to 0.122)	0.954
**Physical activity energy expenditure (kJ/kg/day)**																	
FU1: n=724 (n=335, baseline adjusted), n=658 (n=310, further adjusted); FU2: n=571 (n=253, baseline adjusted), n=520 (n=237, further adjusted)	96.43 (23.16)	91.70 (23.71)	79.66 (22.26)		94.08 (24.38)	91.27 (25.42)	78.60 (22.43)		−0.866 (−6.811 to 5.079)	0.708	0.001 (−5.745 to 5.747)	0.999		−1.762 (−7.007 to 3.482)	0.387	−0.224 (−5.344 to 4.896)	0.910
**Sedentary time (hrs/24 hrs)**																	
FU1: n=720 (n=334, baseline adjusted), n=654 (n=310, further adjusted); FU2: n=575 (n=54, baseline adjusted), n=524 (n=239, further adjusted)	14.42 (1.88)	14.01 (2.12)	15.86 (1.86)		14.57 (1.78)	14.08 (2.20)	15.73 (1.94)		−0.045 (−0.610 to 0.521)	0.839	0.186 (−0.443 to 0.814)	0.447		0.156 (−0.384 to 0.697)	0.456	0.287 (−0.368 to 0.941)	0.260
**MVPA time (mins/24 hrs)**††																	
FU1: n=720 (n=334, baseline adjusted), n=654 (n=310, further adjusted); FU2: n=575 (n=254, baseline adjusted), n=524 (n=239, further adjusted)	57.91 (42.52-85.90)	62.07 (38.80-102.97)	40.79 (31.47-57.19)		59.47 (42.80-81.53)	59.80 (40.91-96.95)	44.36 (32.85-67.94)		−1.310 (−11.843 to 9.224)	0.749	−3.332 (−10.706 to 4.042)	0.245		−3.939 (−16.561 to 8.682)	0.421	−4.314 (−12.697 to 4.070)	0.185
**Achieving ≥60 mins MVPA/24 hs**¶¶																	
FU1: n=720 (n=334, baseline adjusted), n=654 (n=310, further adjusted); FU2: n=575 (n=254, baseline adjusted), n=524 (n=239, further adjusted)	228 (46.44)	207 (52.27)	70 (22.80)		276 (49.55)	234 (49.79)	120 (30.53)		0.041 (−0.085 to 0.207)	0.446	−0.068 (−0.166 to 0.096)	0.215		0.005 (−0.101 to 0.140)	0.911	−0.067 (−0.165 to 0.096)	0.219
**Systolic blood pressure (mm Hg)**																	
FU1: n=1100 (n=513, baseline adjusted), n=778 (n=369, further adjusted); FU2: n=996 (n=447, baseline adjusted), n=771 (n=325, further adjusted)	95.67 (9.04)	95.35 (8.78)	96.98 (8.30)		98.10 (10.06)	95.29 (8.22)	97.75 (8.21)		0.624 (−1725 to 2.973)	0.494	0.310 (−1.528 to 2.148)	0.664		0.931 (−1.307 to 3.169)	0.284	0.577 (−1.431 to 2.584)	0.459
**Diastolic blood pressure (mm Hg)**																	
FU1: n=1100 (n=513, baseline adjusted), n=778 (n=369, further adjusted); FU2: n=996 (n=447, baseline adjusted), n=771 (n=325, further adjusted)	62.18 (7.99)	62.08 (7.81)	63.29 (7.46)		64.21 (8.59)	62.19 (7.43)	63.50 (7.34)		0.335 (−1.721 to 2.392)	0.675	0.482 (−1.570 to 2.533)	0.545		0.945 (−1.247 to 3.137)	0.267	0.517 (−1.605 to 2.639)	0.530
PedsQL total score***																	
FU1: n=1171 (n=538, baseline adjusted), n=817 (n=375, further adjusted; FU2: n=1055 (n=477, baseline adjusted), n=755 (n=346, further adjusted)	71.74 (60.87-82.61)	76.09 (65.22-84.78)	82.61 (71.74-89.13)		73.91 (60.87-82.61)	76.09 (65.22-84.78)	80.43 (71.74-89.13)		−0.630 (−4.385 to 3.124)	0.665	1.248 (−2.301 to 4.796)	0.365		−0.437 (−4.271 to 3.398)	0.769	1.246 (−1.815 to 4.307)	0.294
**PedsQL physical functioning score**																	
FU1: n=1171 (n=538, baseline adjusted), n=817 (n=375, further adjusted; FU2: n=1056 (n=476, baseline adjusted), n=754 (n=346, further adjusted)	73.06 (18.07)	77.79 (16.28)	83.71 (13.86)		74.87 (17.26)	78.86 (15.14)	84.18 (12.85)		−0.649 (−4.006 to 2.708)	0.618	0.118 (−3.411 to 3.646)	0.932		−0.191 (−3.498 to 3.116)	0.882	0.704 (−2.557 to 3.965)	0.578
**PedsQL psychosocial functioning score**																	
FU1: n=1170 (n=538, baseline adjusted), n=817 (n=375, further adjusted); FU2: n=1054 (n=476, baseline adjusted)) n=754 (n=345, further adjusted)	69.47 (17.95)	71.27 (16.58)	77.52 (14.40)		69.28 (18.19)	72.10 (15.81)	76.27 (14.96)		−0.661 (−3.798 to 2.475)	0.587	1.593 (−1.598 to 4.784)	0.198		−0.679 (−3.352 to 1.993)	0.513	1.468 (−1.480 to 4.415)	0.200
**PedsQL emotional functioning score**																	
FU1: n=1171 (n=538, baseline adjusted), n=817 (n=375, further adjusted); FU2: n=1055 (n=477, baseline adjusted), n=755 (n=346, further adjusted)	73.36 (22.20)	75.88 (21.02)	83.42 (18.11)		71.68 (23.05)	75.75 (20.67)	81.57 (18.86)		−0.045 (−4.236 to 4.147)	0.978	1.972 (−1.766 to 5.710)	0.174		0.115 (−3.954 to 4.184)	0.942	2.021 (−1.745 to 5.787)	0.167
**PedsQL social functioning score**																	
,FU1: n=1169 (n=537, baseline adjusted), n=816 (n=374, further adjusted); FU2: n=1053 (n=475, baseline adjusted), n=753 (n=344, further adjusted)	67.72 (22.34)	70.89 (20.52)	76.72 (18.31)		68.60 (21.71)	72.39 (19.39)	75.81 (18.91)		−1.134 (−4.634 to 2.366)	0.404	0.993 (−2.517 to 4.503)	0.466		−1.137 (−4.193 to 1.918)	0.338	1.089 (−2.305 to 4.483)	0.409
**PedsQL school functioning score**																	
FU1: n=1167 (n=538, baseline adjusted), n=814 (n=375, further adjusted); FU2: n=1052 (n=475, baseline adjusted), n=752 (n=345, further adjusted)	67.35 (21.72)	67.04 (20.03)	72.40 (17.50)		67.54 (21.56)	68.07 (18.72)	71.42 (18.21)		−0.810 (−4.533 to 2.912	0.575	1.698 (−2.181 to 5.557)	0.260		−0.876 (−4.331 to 2.579)	0.514	1.447 (−2.077 to 4.971	0.290
**Kidscreen-52 bullying**																	
FU1: n=1156 (n=533, baseline adjusted), n=806 (n=370, further adjusted; FU2: n=1047 (n=475, baseline adjusted), n=749 (n=343, further adjusted)	11.74 (3.20)	13.21 (6.95)	14.30 (7.92)		12.05 (2.97)	14.22 (10.14)	14.05 (6.28)		−1.101 (−2.655 to 0.453)	0.068	0.594 (−0.482 to 1.671)	0.155		−0.544 (−1.930 to 0.842)	0.312	0.359 (−0.799 to 1.516)	0.425
**Body image satisfaction score**																	
FU1: n=1149 (n=533, baseline adjusted), n=805 (n=372, further adjusted); FU2: n=1044 (n=476, baseline adjusted), n=748 (n=344, further adjusted))	1.54 (1.39)	1.37 (1.17)	1.19 (1.04)		1.56 (1.40)	1.27 (1.11)	1.11 (0.96)		0.041 (−0.168 to 0.251)	0.611	0.049 (−0.132 to 0.229)	0.487		0.015 (−0.168 to 0.216)	0.847	−0.024 (−0.185 to 0.137)	0.700

MVPA=moderate to vigorous physical activity; PedsQL=pediatric quality of life inventory; BMI=body mass index; FU1=1st follow-up; FU2=2nd follow-up.

* Adjusted for baseline outcome.

† Adjusted for baseline outcome, baseline pupil level covariates: (sex, ethnicity, deprivation (index of multiple deprivation score for home postcode), 24 hour total energy intake, physical activity energy expenditure), and baseline school level covariates (size (number of pupils on roll), % school population South Asian, % school population Black African-Caribbean, % free school meal eligibility).

‡ Included as primary outcomes in trial registry information.

§ Adjusted for baseline body mass index z score.

¶ Inverse transformation used.

** Sum of four skinfolds included summation of biceps, subscapular, suprailiac, and triceps.

†† Transformed through natural logarithm.

‡‡ Transformed through square root.

§§ Adjusted for total grams of fruit and vegetables consumed in 24 hours.

¶¶ Adjusted for minutes of moderate to vigorous PA per 24 hours.

*** Transformed through squaring.

*Anthropometric measurements*—these are included as primary outcomes in trial registration information (see “Changes to methods from trial registration stage”). In the intervention arm compared with control arm, the baseline adjusted risk difference in the proportion of children who were overweight or obese was −0.013 (99% confidence interval −0.075 to 0.071, P=0.66) and 0.002 (−0.068 to 0.093, P=0.95) at 15 and 30 months, respectively. The mean difference in sum of skinfolds, waist circumference z score, and body fat percentage were all non-significant, but slightly favoured the control group.

*Diet, physical activity, and blood pressure*—the mean differences in total daily energy intake, physical activity energy expenditure, and systolic and diastolic blood pressures between groups were inconsistent in direction and statistically non-significant at both follow-ups.

*Longer term clinical effectiveness*—among group 1 school participants who were followed up at 39 months (488 pupils (246 controls), 27 schools (14 controls)), the mean BMI z score was lower in the intervention arm compared with control arm in the baseline adjusted model (mean difference −0.20, 99% confidence interval −0.46 to 0.05, P=0.04) and further adjusted models (−0.18; −0.39 to 0.03, P=0.03). We were not aware of any contextual or intervention delivery aspects that differed between the groups. To investigate why the intervention appeared more effective at this later time point, we undertook post hoc analysis to consider whether schools recruited in group 1 differed from those in group 2, both in characteristics (see appendix, table A1) and in outcomes at earlier time points (see appendix, table A2). This showed a noticeable imbalance in baseline adiposity between arms in group 2 schools and baseline differences in ethnicity, deprivation, and adiposity between group 1 and group 2 schools. There was a significant interaction in the effect of the intervention on the primary outcome between groups (P=0.001 (first follow-up) and P=0.02 (second follow-up) in the partially adjusted model). Analysis of outcomes by school group showed a statistically significantly lower BMI z score in the intervention arm compared with control arm at first follow-up in group 1 schools (mean difference –0.23, 95% confidence interval −0.35 to −0.12, P<0.01 for baseline adjusted model), which was maintained through to the third follow-up (although no longer statistically significant at the 1% level). In contrast there was no significant difference between arms at any time point in group 2 schools (see appendix, table A2).

*Harms*—quality of life, as total score or subdomains, social acceptance, or dissatisfaction with body image did not differ significantly between arms at any time. Thus we found no evidence of harm from the intervention.

*Subgroup and sensitivity analyses*—all subgroup analyses (by ethnicity, sex, socioeconomic or weight status, and fidelity of implementation) and sensitivity analyses were consistent with the main analyses and did not change any conclusions (results not shown).

*Process evaluation*—detailed results from the process evaluation are reported separately.^17^ ^18^ ^19^ Briefly, the intervention was generally well implemented, although no school delivered all components completely as intended. The scores developed to represent overall fidelity of programme implementation show that just under half the schools (12/26) achieved at least 75% of the maximum possible score and only five schools failed to achieve at least 65% of that maximum. Teachers found the daily physical activity intervention component the most challenging to deliver, with only four of 26 schools (17%) achieving high implementation fidelity for that component and 58% of schools (15/26) allocated to the low implementation fidelity group. In contrast, 42% (11/26) and 65% (17/26) of schools achieved high implementation fidelity for the cooking workshop and Villa Vitality components, respectively, with classification to the low implementation fidelity group for 42% (11/26) (cooking workshop) and 27% (7/26) (Villa Vitality). However, despite some challenges to implementation, the interviews and focus groups indicated that the programme was often well received both by teachers^19^ and by parents and children (see box).^20^

**Quotes about the programme**

It was fantastic and combining the sport and the nutrition was brilliant (teacher)
There’s no doubt about it they’ve loved it, yeah . . . so it’s been really good for them and that’s what it’s all about really isn’t it (teacher)
It’s good to have it reinforced I think from somebody other than your parents, sometimes if your teacher says it, it’s true! (parent)
She’s willing to try more fruits and vegetables, that’s what I’m pleased with probably more, before she was quite picky with what she’d have, but now she is willing to try new things (parent)
I teached my mum how to cook it when we cooked in Aston Villa. And I chop a bit at home because I learned how to chop at Aston Villa (child)
Because I’ve done my exercise I can think harder and try (child)

## Discussion

We found no overall evidence of improvement in the primary outcomes of reduction in body mass index (BMI) z scores at 15 and 30 months after a childhood obesity prevention programme delivered through schools and targeting 6 and 7 year olds. However, confidence intervals did not exclude between arm differences in BMI z score of 0.125, thought to be clinically important for prevention. The intervention did not have any effects on secondary anthropometric, behavioural, or clinical outcomes, and there were no differential effects in prespecified subgroups. A clinically significant difference in BMI z score in favour of the intervention was seen in the first cohort of schools recruited that had data available at 39 months. Subsequent post hoc analysis suggests this may have been a cohort effect, with evidence of effectiveness in group 1 schools at all time points but no effect seen in group 2 schools at any time point. The outcomes used to assess harm did not differ between the groups.

### Strengths and weaknesses of this study

The WAVES study is a large childhood obesity prevention trial within a socioeconomically and ethnically diverse population, with sufficient sample size to assess the primary outcome. Phased development of the 12 month multicomponent intervention was guided by the Medical Research Council framework for complex interventions,^9^ ^21^ including a successful feasibility trial.^10^ The intervention comprised elements identified as promising in systematic reviews^5^ ^6^ and incorporated a range of behaviour change techniques, including those associated with positive outcomes in previous childhood obesity prevention trials.^22^ Outcomes were assessed with mainly objective measurements, using validated instruments and standardised protocols. Loss to follow-up was relatively small, with 80% of pupils retained to the second follow-up, and loss of one school. A prespecified analysis plan took account of clustering, and the findings were robust to a range of sensitivity analyses. This was also one of few trials that undertook longer term follow-up (39 months) to assess sustainability of intervention effects. Comprehensive process evaluation (described in more detail elsewhere^16^) helped to contextualise the findings and to interpret the results.^17^

Nevertheless, there were also several limitations. Parental consent for study measurements being obtained for only 60% of eligible children could introduce selection bias; however, a pupil level comparison of demographic characteristics (sex, ethnicity, deprivation) between those with and those without consent did not show any major differences. The balancing algorithm to allocate schools was based on whole school (cluster) level data. However, within clusters, only children from one year group were included, and just over half of those consented to study measurements. There was notable baseline imbalance between arms in the group 2 cohort (with the intervention arm having greater adiposity than the control arm), which, despite the use of adjustment methods, may have attenuated the main results. Statistical adjustment assumes a common linear relation between covariates and outcome in all clusters, and misspecification of the model may lead to both under-adjustment and over-adjustment. Baseline imbalance is a known limitation of cluster trials and can best be overcome with recruitment of larger numbers of clusters. Although follow-up to 30 months was in all groups, longer term follow-up (to 39 months) was limited to a subset of participating schools. The Child And Diet Evaluation Tool (CADET) provided a quick, practical dietary assessment tool with relatively low respondent burden,^24^ ^25^ resulting in useable data from approximately 85% of children at baseline (81% first follow-up, 82% second follow-up). However, estimates of dietary intake may not reflect habitual intake, there was a risk of misreporting,^26^ and there may have been seasonal variation^27^ between data collection periods. Usable data on physical activity were available for 76% of children at baseline (60% first follow-up, 52% second follow-up). These are similar to the rates achieved in other such studies.^28^

### Comparison with other studies

Our results build on the findings of previous reviews and address limitations in previous childhood obesity prevention trials. Two systematic reviews suggested that there was moderate^6^ to strong^5^ evidence of effectiveness of school based interventions in preventing childhood obesity, although heterogeneity of interventions, variable design quality, and lack of longer term follow-up limit interpretation. A meta-analysis showed that the summary magnitude of effect on BMI z score compared with the control was −0.15 units,^5^ which is smaller than the effect size used for estimating sample size in our trial. Nevertheless, the WAVES study was larger than the 21 previous obesity prevention trials with low risk of bias included in the meta-analysis (n=9 to 574). Since the publication of the reviews, findings from another UK cluster randomised controlled trial, the Active for Life Year 5 (AFLY5) including more than 2000 children from 60 schools are available.^28^ The trial primarily attempted to influence activity levels and fruit and vegetable consumption, although it also reported on adiposity outcomes. The intervention was curriculum based, focusing on educational approaches rather than the more experiential skills based intervention in the WAVES study. In contrast with our trial, the target population was children at the end of the primary school years, when rates of obesity have already increased substantially, and included few children from minority ethnic groups and more deprived areas. Nevertheless, similar to our findings, there was no evidence of an intervention effect on behavioural or weight outcomes at 12 months.

### Interpretation of the findings

The balance of components, intensity, and behaviour change strategies used to deliver the intervention may have contributed to the absence of evidence of effect on the primary outcomes in WAVES and other trials. Although fidelity of implementation for the WAVES study intervention programme was reasonably high overall, no school delivered all components completely per protocol, and a few schools failed to deliver some or all of the components. This may have attenuated any effect.^29^ In addition, owing to competing demands on teachers, components that required greater teacher input tended to be less well implemented and this was the main explanation for differences in fidelity between components. This suggests that delivery of a more intensive teacher led intervention in a school setting would not be feasible without additional resources. Educational and experiential interventions of longer duration that are embedded within a whole school setting are likely to be prohibitively costly and complex to evaluate using clinical trial methods. The intervention was developed on the basis of promising strategies in trials published before the feasibility study (about 10 years before the definitive trial). As a result, strategies such as those based on behavioural economics aimed at altering the social and physical environment were not included as part of the intervention. Although the findings from the feasibility study suggested the WAVES intervention was promising, intervention delivery for the trial and subsequent follow-up measurements took place some years later, during which time wider environmental changes might have diluted any effects. Researcher contact with schools during the feasibility study was also much greater, but this was not replicable in the definitive trial with a larger number of schools and would not be implementable outside of a trial setting. Methodological limitations with baseline imbalance may have also contributed to the observed findings with heterogeneity of effect between schools. However, even the cohort effect observed in group 1 schools was small, suggesting that childhood obesity prevention is unlikely to be achieved by schools alone. While school is an important setting for influencing children’s health behaviour, and delivery of knowledge and skills to support healthy lifestyles is one of its mandatory functions, wider influences from the family, community, media, and the food industry must also be considered. The qualitative data from teachers^19^ and parents,^20^ collected as part of our process evaluation, support the possibility that these wider influences have a greater effect than any school based intervention. A metasynthesis of qualitative studies exploring the role of primary schools in preventing childhood obesity highlighted the need for schools, parents, and government to work together to promote healthy lifestyles in children and to support activities in the school setting.^30^

### Conclusions

The multicomponent WAVES study intervention, which was feasible to deliver and for which there was no evidence of harm, did not result in a statistically significant difference in BMI z score overall, and there was no evidence of effect on measured diet or physical activity levels in children. Although wider implementation of this intervention cannot be recommended for obesity prevention, the lower cost components could be considered by schools to fulfil their mandated responsibilities for education on health and wellbeing. Within the context of the wider evidence, it is likely that any effect of school based educational, motivational, and skill centred interventions on obesity prevention is small. Several community based interventions targeting wider environments have also been evaluated recently, using non-randomised experimental designs. Although a few of these have shown evidence of small effects and lower weight gain in children from intervention communities,^31^ ^32^ the findings are not consistent^33^ and need further evaluation. Interventions based on behavioural economics such as nudge theory^34^ also merit further investigation. Even marginal effects may be important within a wider systems approach to obesity prevention, which incorporates multiple agencies and widespread policy change to support healthy behaviours.

**What is already known on this topic**

Comprehensive systematic reviews have suggested that school based interventions could be effective in preventing childhood obesity in high income countries
Heterogeneity in intervention components and outcomes limit practical recommendations
Furthermore, inconsistent findings in relation to differential effects on subgroups, and impact on inequalities, limited data on potential harms, process measures, and long term effects, as well as lack of data on cost effectiveness, restrict interpretation and wider applicability

**What this study adds**

The WAVES study evaluated a theoretically informed, skills based intervention targeting children’s diet and physical activity behaviours through schools and families
It did not result in any meaningful effect on adiposity, dietary intake, or physical activity after 15 or 30 months
Although such interventions can fulfil the responsibility of schools for wider education, without upstream support they are unlikely to halt the childhood obesity epidemic

## Notes

Contributors: All WAVES study trial co-investigators contributed to the development of the design for the WAVES study trial and had contributed to the intervention development as part of the BEACHeS Study. PA wrote the first draft of the paper and all authors contributed to critical revisions. PA, MP, and KKC planned the overall design of the trial. ERL coordinated all aspects of the trial, oversaw data collection, collation, and cleaning, and contributed to data analysis. She also contributed to the first draft of the manuscript. EF contributed to the design of the trial and analysis plan, and AC advised on study instruments and undertook some of the analysis. JJD and KaH contributed to sampling, sample size estimation, and the statistical analysis plan. JM undertook data analysis, supervised by KaH and with support from ERL. UE advised on physical activity measurements and related methods and oversaw the preparation of the physical activity data. JEC advised on dietary assessment and related methods and oversaw the CADET data preparation. KiH led data collection and analysis of dietary data. JLD advised on some of the psychosocial assessment methods. TB, PG, and RB advised on clinical measurement processes. RB and PG advised on aspects related to ethnicity. AD advised on the physical activity intervention component. SP advised on school recruitment and approaches to keeping schools engaged. EM advised on the dietary intervention components. JP advised on process evaluation, and TG designed the detailed methods for this. TG and JLC contributed to data collection and undertook analysis and interpretation of the process evaluation. The final manuscript was read and approved by all authors. PA, ERL, and MP are guarantors.

Ethical approval: The trial was approved by NHS Research Ethics Service Committee West Midlands, The Black Country (NHS REC No 10/H1202/69).

Data sharing: Requests for access to data from the WAVES study should be addressed to the corresponding author at p.adab@bham.ac.uk . All the individual participant data collected during the trial (including the data dictionary) will be available, after deidentification, immediately after publication with no end date. The study protocol has been published. All proposals requesting data access will need to specify how it is planned to use the data, and all proposals will need approval of the trial co-investigator team before data release.

Transparency: The guarantors (PA, ERL, and MP) affirm that the manuscript is an honest, accurate, and transparent account of the study bring reported; that no important aspects of the study have been omitted; and that any discrepancies from the study as planned (and, if relevant, registered) have been explained.
